# Neuropeptide Y2 Receptor (*NPY2R*) Expression in Saliva Predicts Feeding Immaturity in the Premature Neonate

**DOI:** 10.1371/journal.pone.0037870

**Published:** 2012-05-21

**Authors:** Jill L. Maron, Kirby L. Johnson, Jessica A. Dietz, Minghua L. Chen, Diana W. Bianchi

**Affiliations:** 1 Division of Newborn Medicine, Department of Pediatrics, Floating Hospital for Children at Tufts Medical Center, Boston, Massachusetts, United States of America; 2 Division of Genetics, Department of Pediatrics, Floating Hospital for Children at Tufts Medical Center, Boston, Massachusetts, United States of America; 3 Mother Infant Research Institute at Tufts Medical Center, Boston, Massachusetts, United States of America; University of Massachusetts Medical School, United States of America

## Abstract

**Background:**

The current practice in newborn medicine is to subjectively assess when a premature infant is ready to feed by mouth. When the assessment is inaccurate, the resulting feeding morbidities may be significant, resulting in long-term health consequences and millions of health care dollars annually. We hypothesized that the developmental maturation of hypothalamic regulation of feeding behavior is a predictor of successful oral feeding in the premature infant. To test this hypothesis, we analyzed the gene expression of neuropeptide Y2 receptor (*NPY2R*), a known hypothalamic regulator of feeding behavior, in neonatal saliva to determine its role as a biomarker in predicting oral feeding success in the neonate.

**Methodology/Principal Findings:**

Salivary samples (n = 116), were prospectively collected from 63 preterm and 13 term neonates (post-conceptual age (PCA) 26 4/7 to 41 4/7 weeks) from five predefined feeding stages. Expression of *NPY2R* in neonatal saliva was determined by multiplex RT-qPCR amplification. Expression results were retrospectively correlated with feeding status at time of sample collection. Statistical analysis revealed that expression of *NPY2R* had a 95% positive predictive value for feeding immaturity. *NPY2R* expression statistically significantly decreased with advancing PCA (Wilcoxon test p value<0.01), and was associated with feeding status (chi square p value  =  0.013).

**Conclusions/Significance:**

Developmental maturation of hypothalamic regulation of feeding behavior is an essential component of oral feeding success in the newborn. *NPY2R* expression in neonatal saliva is predictive of an immature feeding pattern. It is a clinically relevant biomarker that may be monitored in saliva to improve clinical care and reduce significant feeding-associated morbidities that affect the premature neonate.

## Introduction

The current practice in newborn intensive care units is to have caregivers subjectively assess when a premature infant is ready to feed by mouth. When the assessment is inaccurate, morbidities may include choking, hypoxia, aspiration, oral aversion, and prolonged hospitalization [1]–[3]. These associated morbidities are estimated to cost millions of U.S. health care dollars annually. A barrier to the development of an objective diagnostic assay is the ability to directly assess the complex neuromuscular and neurological sequences required for the development of safe and effective oral feeding skills in the premature neonate [4]–[6]. Furthermore, feeding behavior is the result of a complex interplay of feedback loops, satiety, and cellular signaling that is under the direct control of the hypothalamus [7], [8]. While hypothalamic regulation of feeding behavior is an area of active investigation, such research has frequently been conducted on genetically altered animal models with post-mortem examinations to allow for direct visualization and staining of the hypothalamus and its associated neurological pathways [9]–[12].

Neuropeptide Y (*NPY*) and its family of five receptors are known to be associated with hypothalamic regulation of feeding behavior, metabolism, and energy homeostasis in both rodents and humans [9]–[12]. In particular, knock-out mice for the gene neuropeptide Y 2 receptor, *NPY2R*, exhibit hyperphagia and excessive weight gain [9]. As a result, research on the gene has focused on genotyping analysis for the identification of single nucleotide polymorphisms (SNPs) in obese human subjects [13], [14] and drug development for manipulation of its expression as a novel therapeutic treatment for obesity [15], [16]. Although primarily expressed in the arcuate nucleus of the hypothalamus [17], *NPY2R* may also be found in other tissues including trabecular bone [18], vascular tissue [19], and colonic mucosa [20].

Previously, our laboratory published on the diverse range of gene expression changes occurring in the premature neonate that can be detected in as little as 10 µL of saliva. Specifically, we showed that expression of *NPY2R* in neonatal saliva statistically significantly changed over time as newborns learned to orally feed [21]. This work suggested that the hypothalamus plays a central role in feeding behavior in the premature neonate. Further, through noninvasive salivary gene expression analyses, we can monitor, in real-time, hypothalamic regulation as it directly relates to the learning process of oral feeding. Here, we hypothesized that the physiological hyperphagia and exponential weight gain observed in healthy term newborns is associated with decreased expression of *NPY2R*. Persistence of *NYP2R* in salivary samples would suggest immature hypothalamic regulation, indicative of failed oral feeding trials. In this study, we independently explored *NPY2R*'s expression profile in relation to feeding status and post-conceptual age (PCA), and determined its role as a biomarker in neonatal saliva to objectively predict successful oral feeding in the newborn.

## Results

### Demographics and Sample Characteristics

One hundred and sixteen salivary samples (10 to 50 µL) were collected from 76 newborns with post-conceptual ages (PCAs) ranging from 26 4/7 to 41 4/7 weeks. There were 63 preterm and 13 term infants in this data set. Pertinent clinical information for all subjects is shown in Table 1, while a detailed summary of clinical sequelae for each individual subject is available in Table S1. In general, subjects in the study were either healthy term newborns, or premature infants who had resolution of their acute clinical sequelae and were “feeders and growers” in the NICU prior to discharge home. Of the 76 subjects enrolled in this study, 31 had between two and five salivary samples analyzed, at either different PCAs and/or feeding statuses. The number of samples collected from subjects at each predefined feeding stage, as well as the percentage of infants expressing *NPY2R*, was as follows: Stage 1: no feeds (NPO) (n = 17; PCA 25 3/7 to 36 1/7 weeks; 59% *NPY2R* expression); Stage 2: partial per gastric feeds (PPG) (n = 21, PCA 28 2/7 to 41 3/7 weeks; 57% *NPY2R* expression); Stage 3: full per gastric feeds (FPG) (n = 36, PCA 28 5/7 to 37 5/7 weeks; 67% *NPY2R* expression); Stage 4: partial oral feeds (PPO) (n =  24, PCA 33 5/7 to 41 2/7 weeks; 50% *NPY2R* expression); Stage 5: full oral feeds (FPO) (n = 18, PCA 33 3/7 to 40 2/7 weeks; 17% *NPY2R* expression).

**Table 1:** Pertinent Clinical and Demographic Information.

Feeding Stage	Number of Subjects	PCA (weeks)	Weight (kg)	Summarized Medical Complications of Subjects
1 (NPO)	17	25 3/7–36 1/7	0.73–2.136	Respiratory distress syndrome (RDS), patent ductus arteriosus (PDA), intrauterine growth restriction (IUGR), bronchopulmonary dysplasia (BPD), urinary tract infection (UTI), neonatal abstinence syndrome (NAS), pulmonary valvular stenosis, apnea, hyperbilirubinemia, ABO incompatibility, anemia, anal fissure, choanal atresia, leukocytosis, metabolic acidosis, undescended testicle, multiple gestation
2 (PPG)	21	28 2/7–41 3/7	0.78–3.845	RDS, PDA, BPD, IUGR, apnea, anemia, thrombocytopenia, coagulopathy, hyperbilirubinemia, ABO incompatibility, transient tachypnea, multiple gestation, bacteremia, persistent pulmonary hypertension (PPHN), metabolic acidosis
3 (FPG)	36	28 5/7–37 5/7	0.911–2.215	RDS, IUGR, BPD, right grade I intraventricular hemorrhage (IVH), leukocytosis, acidosis, neutropenia, peripheral pulmonary stenosis, transient tachypnea, multiple gestation, narcotic exposure, bacteremia, polydactyly
4 (PPO)	24	33 5/7–41 2/7	1.445–3.678	RDS, NAS, IUGR, small for gestational age (SGA), anemia, apnea, hypertension, hemangioma, hyperbilirubinemia, multiple gestation, twin-to-twin transfusion, thrombocytopenia, anal fissure, membranous choanal atresia, hypermagnesia
5 (FPO)	18	33 3/7–40 2/7	1.807–3.910	Hyperbilirubinemia, ABO incompatibility, RDS, BPD

### Multiplex Reverse Transcription-quantitative Polymerase Chain Reaction (RT-qPCR) Characteristics

Every effort was made to adhere to the Minimum Information for Publication of Quantitative Real-Time PCR Experiments (MIQE) guidelines [22]. Multiplex RT-qPCR amplification was performed on all extracted total RNA samples for the gene *NPY2R*, along with three reference genes: glyceraldehyde-3-phosphate dehydrogenase (*GAPDH*), tyrosine 3-monoxygenase/tryptophan 5-monooxygenase activation protein, zeta polypeptide (*YWHAZ*), and hypoxanthine phosphoribosyltransferase 1 (*HPRT1*). Reference genes were selected based upon microarray data from previous studies conducted in our laboratory that revealed that each of these genes maintains a relative constant and consistent range of expression across different PCAs. We selected one gene known to be expressed at a relatively high level (*YWHAZ*), one that had an average expression level (*GAPDH*), and one that demonstrated a low level of expression (*HPRT1*) at the limit of our detection level on the multiplex platform.

Prior to multiplex RT-qPCR, three samples were tested simultaneously on both uniplex and multiplex platforms for all genes to assess reaction efficiencies. There was no difference in reaction efficiency between uniplex and multiplex assays. Of the three test samples run in both formats, quantification cycle (Cq) values were within 1 cycle for all genes. Thus, we were confident that running the samples on a multiplex platform would not impact results. The reference gene *YWHAZ* had the highest level of expression in our samples (mean Cq: 27.7), followed by *GAPDH* (mean Cq: 30.3), and *HPRT1* (mean Cq: 36.8). Mean delta Cq and standard deviation values between reference genes for all reactions were as follows: *GAPDH-YWHAZ*: 2.3*+/−*1.85; *HPRT1-GAPDH* 7.2+/−1.83; *HPRT1-YWHAZ* 9.5+/−1.93. These results demonstrate that reaction efficiencies across experiments were similar and reproducible. Of the 116 samples analyzed, 95 had amplification of all three reference genes, while 21 revealed amplification of *GAPDH*, and *YWHAZ* only. Of these 21 samples, six were positive for *NPY2R* expression and 15 had no detectable *NPY2R* in the sample.

### Assay Results

**Figure 1:**
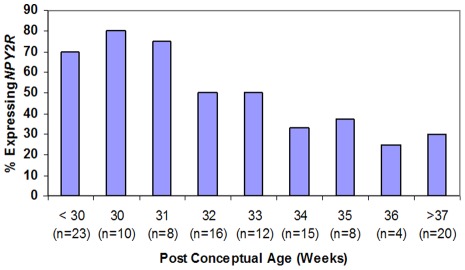
*NPY2R* gene expression and advancing post-conceptual age in weeks (all infants).

**Figure 2:**
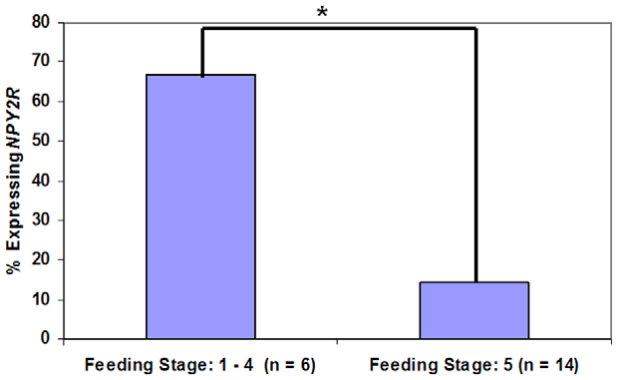
*NPY2R* gene expression and feeding status in term infants.

**Figure 3:**
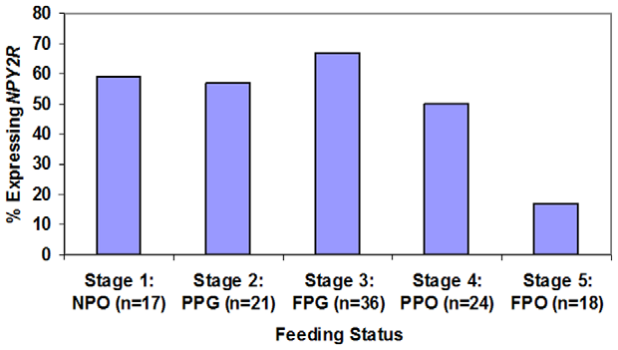
*NPY2R* gene expression and feeding status (all infants).

For the purpose of this study, we performed three separate statistical analyses on the data. The first analysis considered all 116 data points; the second analysis considered only one sample per subject in order to ensure that multiple sampling from an individual was not skewing the data (n = 76); the third analysis removed samples that did not have amplification of all three housekeeping genes in order to eliminate those samples that had a theoretical risk of a false negative result (n = 95). *NPY2R* expression in neonatal saliva for all 116 samples had a 95% positive predictive value (CI: 85%–99%) of an immature feeding pattern with an inability to sustain full oral feeds (Stage 5). The negative predictive value of the assay was 27% (CI: 17%–41%). The sensitivity was 59% (CI: 49%–69%) with 83% specificity (CI: 58%–96%). There was a statistically significant difference between *NPY2R* expression and PCA. Neonates who expressed *NPY2R* were younger than those infants who did not express the gene (p value<0.01) (Figure 1). However, among term infants, there was a statistically significant difference between infants who could and could not orally feed (p value  =  0.037) (Figure 2). Expression of *NPY2R* was associated with feeding status (p value  =  0.013) (Figure 3). As infants matured through the feeding stages, they were less likely to express the gene.

Neither of the two additional statistical analyses altered our primary results. Limiting the data set to include only one salivary sample per subject revealed a positive predictive value of 97% (CI: 85%–100%), with a negative predictive value of 38% (CI: 23%–55%). Sensitivity of the assay was 62% (CI: 50%–75%) with specificity of 93% (CI: 66%–100%). *NPY2R* expression remained statistically significantly associated with advancing PCA (Wilcoxon rank sum test p value<0.01) and feeding status (chi square p value  =  0.004). Further, eliminating the samples that did not have amplification of all three housekeeping genes in order to reduce the potential impact of false negative results had a positive predictive value of 95% (CI: 84%–99%), negative predictive value of 33% (CI:20%–50%), with a sensitivity of 67% (CI: 55%–77%) and specificity of 81% (51%–95%). *NPY2R* expression was marginally statistically significantly associated with advancing PCA (Fisher's exact test p value  =  0.054). There remained a nonrandom association between *NPY2R* expression and feeding status (chi square p value  =  0.076).

## Discussion

Our results demonstrate that detection of *NPY2R* in neonatal saliva has a 95% positive predictive value for determining that an infant cannot sustain full oral feeds, implying that this gene plays an important role in neonatal feeding behavior. Our data suggest that *NPY2R* is a novel biomarker that could be used as part of an objective assay prior to the introduction of oral feeds, particularly for those infants at risk for aspiration, hypoxia, and long-term feeding aversion.

Although the precise molecular mechanisms by which *NPY2R* influences neonatal feeding are currently unknown, our data demonstrate that expression of *NPY2R* is independent of the presence of enteral nutrition if given by catheter. We were only able to detect down-regulation of the gene once infants were able to take at least some feeds by mouth. Thus, stimulation of the gastrointestinal tract alone is not enough to cause decreased gene expression. Similarly, although *NPY2R* expression is statistically significantly negatively correlated with advancing PCA, here too, it appears that the PCA of the infant is not the sole driving force behind *NPY2R* expression. Some subjects in this study could successfully orally feed prior to 34 weeks' PCA, while others at 41 weeks' PCA could not. In fact, there was a statistically significant difference in *NPY2R* expression between infants who could and could not orally feed at term gestation. Thus, while advancing gestational age correlates with decreased *NPY2R* expression, the relationship is complex and not mutually inclusive. Of note, neonates in this study had a wide range of ages and clinical sequelae. The deliberate incorporation of a diverse patient population was essential to determine the applicability and accuracy of *NPY2R* as a diagnostic salivary biomarker.

We believe that *NPY2R*'s relatively weak negative predictive power confirms the complexities of oral feeding in the newborn. The physical limitations of some infants, including lung disease, reliance on oxygen and poor gastrointestinal motility, may prohibit neonates from sustaining oral feeds who otherwise appear to have appropriate hypothalamic signaling. In addition, subjective assessments by the caregivers that infants could not safely feed and therefore did not allow the neonates to attempt oral feeds may have skewed the data. Thus, future work must be performed to identify additional biomarkers in neonatal saliva that will incorporate all aspects of feeding in the neonate, including maturation of oral musculature and innervation.

The potential to monitor hypothalamic regulation of feeding behavior through salivary gene expression analysis is also a major conclusion of this study. Real-time gene expression of *NPY2R* may be obtained noninvasively, in as little as 10 µl of saliva, from human subjects. We speculate that our ability to readily detect *NPY2R* in neonatal saliva is a direct result of the permeability of the arcuate nucleus to the blood brain barrier [17]. As a filtrate of blood, saliva has been called a ‘window into the body’ with a biomarker profile similar to plasma [23].

The analysis of *NPY2R* gene expression as it relates to oral feeding in the premature neonate provides a window into developmental biology that has the potential to transcend into other areas of medicine. The early newborn period represents a unique time in the human lifespan when hyperphagia and exponential weight gain is normal. The average newborn will gain 200% of his or her birth weight by one year of age. Our results suggest that developmentally, the human neonate down-regulates *NPY2R* expression in order to demonstrate the necessary hyperphagia required for this exponential growth. We speculate that understanding the normal developmental expression of the gene, combined with its ease of monitoring in human saliva, has the potential to help elucidate pathological conditions, including obesity.

Previous studies on neonatal oral feeding almost exclusively focused on oral motor skills, the suck-swallow reflex, neurodevelopment and neuronal integration [4], [5]. Here, we speculate that in addition to these essential developmental milestones, satiety, as regulated, in part, by *NPY2R* expression, also plays a key role in oral feeding success. This is based both upon the numerous published reports linking *NPY2R* expression with hunger, satiety and feeding behavior in both humans and animals [9]–[14], and our previous work on the neonatal salivary transcriptome demonstrating that *NPY2R* had statistically significantly gene expression changes over time as infants matured and learned to orally feed [21]. By demonstrating that detection of *NPY2R* in neonatal saliva is predictive of failed oral feeding trials, we have substantiated our hypothesis that *NPY2R* does play a role in the attainment of successful newborn oral feeding.

In conclusion, *NPY2R* is a predictive novel biomarker in neonatal saliva that may be monitored noninvasively in order to objectively determine when an infant can safely feed by mouth. While future research is needed to further elucidate the precise molecular role of *NPY2R* in relation to human newborn feeding, its presence in neonatal saliva nevertheless remains 95% predictive of an immature feeding pattern and an inability to sustain full oral feeds. This work lays the foundation for the development of an objective diagnostic test to accurately and safely predict oral readiness to feed in this vulnerable population, significantly improving clinical care, and reducing millions of dollars of health care costs associated with feeding morbidities in the premature neonatal population.

## Materials and Methods

### Ethics Statement

This study was approved by the Tufts Medical Center Institutional Review Board. Written parental consent was obtained for all neonatal subjects enrolled.

### Demographics and Sample Characteristics

Salivary samples were collected from premature and term neonates with a diverse range of clinical sequelae at various feeding stages during hospitalization: Stage 1: no feeds (NPO); Stage 2: partial gastric feedings (PPG); Stage 3: full gastric feedings (FPG); Stage 4: partial oral feeds (PPO); Stage 5: full oral feeds (FPO). Infant feeding stage at time of collection was determined solely by the caregivers and was not influenced by study participation. The number of samples collected from a subject was dependent upon their clinical course. In general, premature infants had more than one salivary sample obtained as they matured through the feeding process, while healthy term infants had only one sample obtained at feeding stage 5, FPO. Although salivary samples were collected prospectively from all enrolled subjects, the correlation between salivary *NPY2R* gene expression and feeding status was made retrospectively once all samples were obtained and analyzed.

### Salivary Collection and mRNA Extraction

All salivary samples were collected and processed with previously described standardized techniques that aim to simulate routine bedside care of the neonates [24]. Samples were stored at 4°C for a minimum of 48 hours prior to total RNA extraction with the use of the RNA Protect Saliva Mini Kit (Qiagen, Valencia, CA USA) per manufacturer's instructions. On column DNase digestion was performed on all samples to limit DNA contamination. Final elution volume was approximately 14 µL. Samples were stored at −80°C pending further analysis.

### Multiplex qRT-PCR

All RT-qPCR experiments were performed on the Life Technologies 7900 instrument with the use of the Path-ID™ Multiplex One-Step RT-PCR Kit (Life Technologies, Carlsbad, CA USA). Standard stock sequences of reference genes were provided by Life Technologies and were VIC labeled, primer limited. They were as follows: *GAPDH*-VIC (Hs03929097), *HPRT1*-VIC (Hs01003267), and *YWHAZ*-VIC (Hs03044281). Gene sequences for *NPY2R* were custom made with the use of Primer Express Software v 1 to ensure optimal G-C content and melting temperatures. The custom sequence for *NPY2R* (Sequence accession number: NM_000910.2) was as follows: Forward Primer: GGC TTT CCT CTC GGC CTT C; Reverse Primer TGT CAC GGA CAC CTC AGA GTG; Probe 6FAM-CTG TGA GCA GCG GTT GGA TGC CAT-TAMRA. The amplicon is located at base pairs 1496 to 1563 of the gene and to the best of our knowledge, contains no SNPs. There are only two known exons for the gene *NPY2R*. The entire amplicon used in this study was contained within one exon.

For each salivary sample, *NPY2R* was run in triplicate, multiplexed one time each with the three reference genes. Negative controls with nuclease-free water were performed on each plate. The total volume for each reaction was 25 µL including 2.5 µL of template in each well. The thermal cycle profile for all reactions was as follows: 48°C for 10 minutes, 95°C for 10 minutes, followed by 40 cycles of PCR with a 15 second denaturing cycle at 95°C, followed by 45 seconds of annealing and extension at 60°C.

For the purposes of this study, only the expression of *NPY2R* in neonatal saliva was considered. The gene was considered expressed if it amplified along with ≥2 of the reference genes. Similarly, *NPY2R* was considered not expressed if it did not amplify in the presence of ≥2 of the reference genes. Lacking any reference values for salivary *NPY2R*, it is difficult to provide a clinical interpretation based upon normalized relative quantitative values. Therefore, we analyzed the data in the context of expression of the gene in order to provide our most accurate and biologically relevant assessment. The binary nature of the assay makes it well suited for the development of a rapid diagnostic assay. Finally, average Cq for each reference gene was calculated, along with mean delta Cq and standard deviation values between housekeeping genes to assess efficiencies and variability between reactions.

### Statistical Analyses

Statistical analyses included the Fisher's exact test to determine the relationship of *NPY2R* expression and gestational age, as well as a Wilcoxon rank sum test to compare gestational age in the groups of infants that did or did not express *NPY2R* (Figure 1). Chi-square test was used to assess the association between *NPY2R* gene expression and feeding status (Stages 1–5) (Figure 3). We further examined *NPY2R* expression at term gestation (≥37 weeks' gestation) with the use of a Fisher's exact test to compare expression of the gene in saliva between infants who could and could not successfully feed (Figure 2). Sensitivity, specificity, negative and positive predictive values, along with each respective confidence interval, of the assay were then calculated. Additional statistical analyses were performed to (1) limit the analysis to those samples that had amplification of all three reference genes and to (2) ensure that repeat measures from the same individuals in this study did not skew the data. In the latter analysis, we used only one sample per infant, determined by a random computer number generator to reduce the risk of bias.

## Supporting Information

Table S1 **Complete subject demographic and clinical information.**
(XLSX)
